# *Echinacea purpurea* (L.) Moench treatment of monocytes promotes tonic interferon signaling, increased innate immunity gene expression and DNA repeat hypermethylated silencing of endogenous retroviral sequences

**DOI:** 10.1186/s12906-021-03310-5

**Published:** 2021-05-12

**Authors:** Ken Declerck, Claudina Perez Novo, Lisa Grielens, Guy Van Camp, Andreas Suter, Wim Vanden Berghe

**Affiliations:** 1 Laboratory of Protein Chemistry, Proteomics and Epigenetic Signaling (PPES), Department of Biomedical Sciences, University of Antwerp (UA), Antwerp, Belgium; 2 Center of Medical Genetics, Department of Biomedical Sciences, University of Antwerp (UA) and University Hospital Antwerp (UZA), Antwerp, Belgium; 3 A. Vogel Bioforce AG, Roggwil, Switzerland

## Abstract

**Background:**

Herbal remedies of *Echinacea purpurea* tinctures are widely used today to reduce common cold respiratory tract infections.

**Methods:**

Transcriptome, epigenome and kinome profiling allowed a systems biology level characterisation of genomewide immunomodulatory effects of a standardized *Echinacea purpurea (*L.) Moench extract in THP1 monocytes.

**Results:**

Gene expression and DNA methylation analysis revealed that Echinaforce® treatment triggers antiviral innate immunity pathways, involving tonic IFN signaling, activation of pattern recognition receptors, chemotaxis and immunometabolism. Furthermore, phosphopeptide based kinome activity profiling and pharmacological inhibitor experiments with filgotinib confirm a key role for Janus Kinase (JAK)-1 dependent gene expression changes in innate immune signaling. Finally, Echinaforce® treatment induces DNA hypermethylation at intergenic CpG, long/short interspersed nuclear DNA repeat elements (LINE, SINE) or long termininal DNA repeats (LTR). This changes transcription of flanking endogenous retroviral sequences (HERVs), involved in an evolutionary conserved (epi) genomic protective response against viral infections.

**Conclusions:**

Altogether, our results suggest that Echinaforce® phytochemicals strengthen antiviral innate immunity through tonic IFN regulation of pattern recognition and chemokine gene expression and DNA repeat hypermethylated silencing of HERVs in monocytes. These results suggest that immunomodulation by Echinaforce® treatment holds promise to reduce symptoms and duration of infection episodes of common cold corona viruses (CoV), Severe Acute Respiratory Syndrome (SARS)-CoV, and new occurring strains such as SARS-CoV-2, with strongly impaired interferon (IFN) response and weak innate antiviral defense.

**Supplementary Information:**

The online version contains supplementary material available at 10.1186/s12906-021-03310-5.

## Background

Distinct species of the plant genus *Echinacea* have traditionally been used in North America against infectious diseases and wounds [1, 2]. Currently, a wide variety of *Echinacea* preparations are used world-wide as complementary herbal remedy to improve the immune response to protect against common cold symptoms and influenza infections. Of all *Echinacea* species, *Echinacea purpurea* (purple coneflower) is the most popular variety used in Western countries. Different *Echinacea purpurea* extracts (different species, plant parts, manufacturing) or derived compounds showed antioxidant, antibacterial, antifungal, antiviral and mosquitocidal activities in cell culture experiments [3], although absolute comparisons between studies with different preparations remain difficult [4, 5]. Complex immunomodulatory actions of *Echinacea* have been described including both pro- and anti-inflammatory effects [2, 3, 6]. The compounds that contribute to these activities are alkylamides, glycoproteins, polysaccharides and caffeic acid derivates that may act independently or in synergy [1, 3, 7–9].

In this study, we evaluated Echinaforce®, a commercially registered herbal medicinal tincture of *Echinacea purpurea* (L.) Moench (A.Vogel Bioforce, Switzerland) in several European countries including Switzerland, Austria, UK, Spain, Netherland, Denmark, Finland, Sweden, Slovenia, as well as Canada. The tincture contains 5% root extract and 95% herb extract following extraction with 65% ethanol V/V. Echinaforce® phytochemicals reveal immune modulatory, anti-inflammatory, anti-bacterial, anti-viral and anti-parasitic activity [9–21]. Clinical efficacy could be shown with different batches in acute treatment [22] or for prevention [23] of respiratory tract infections. A 4-month randomized, double blind, placebo-controlled study (*n* = 755 subjects, of which 376 received placebo) on the safety and efficacy of Echinaforce® to prevent common cold symptoms, showed significantly less cold episodes and of shorter duration as well as lower infection recurrence rate in the Echinaforce® treated versus placebo treated group [23]. Moreover, no differences between placebo and Echinaforce® group were reported in relation to health risk and safety [23]. Despite the promising immune potentiating properties of Echinaforce®, the responsible molecular targets have only partially been identified, such as the cannabinoid receptor 2 (CB2) [7, 19, 24], the cAMP, p38/MAPK and JNK signaling pathways, as well as NF-κB and ATF2/CREB1 transcription factors. To further clarify its mode of action, we applied a system biology approach by integrating genomewide transcriptome, epigenome and kinome signaling profiles of THP1 monocytes treated with Echinaforce®.

## Methods

### Cell lines and treatments

Echinaforce® (batch nr. 040070, A. Vogel Bioforce AG, Roggwil, Switzerland) is a standardized preparation obtained by ethanol extraction of freshly harvested *Echinacea purpurea* herb and roots (95:5). The extract Echinaforce® itself is strictly produced under GMP conditions and tested therefore on different levels (seed, plant, extract, tablet, etc.) thoroughly in the same manner as an allopathic remedy since it is a registered product in Europe. The plant has been identified taxiconomically and also with a DNA test. The same seeds of this plant have been used for more than 50 years (since 1955) in the company A. Vogel to produce the standardized test item Echinaforce®. This means that end of year a part of the *Echinacea* plant is used to take the seeds in late autumn to use them for plantation next spring then. Since the majority of the *Echinacea* cultivation is in the vicinity of A. Vogel AG in Roggwil (Switzerland) no adulteration with other plants takes place. According to Good Agricultural Practice, for every batch used for for production of the standardized extract, the plant species is visually verified by an expert before it is released for production of the registered medicinal herbal extracts. The main basis for releases of any batch of Echinaforce® is the HPLC fingerprint, TLCs and a minimum amount of the alklymide tetraen as a marker substance. The composition of marker compounds like alkylamides (i.e. those compounds known to characterize this species of Echinacea) was described previously [3, 6, 25]. With this strategy, A. Vogel can guarantee that every batch is similar in its constituents and its activity profile. Extended research on pharmacological activity with different batches have been carried out by the company showing consistent activity in in vitro settings (antiviral, immunemodulatory activity). In contrast to pressed juice extracts, Echinaforce® extract does not contain polysaccharides which are known to stimulate the immune system nonspecifically [26–29]. The alcohol concentration of Echinaforce® tincture extract was 65% v/v and solvent controls have been included in all experimental in vitro experiments to rule out nonspecific effects. In addition, the preparation was free of detectable endotoxin as determined by means of a commercial assay kit with a lower limit of detection 0.1 unit/ml (Lonza Walkersville Inc., MD).

THP1 cells were grown in RPMI-1640 medium supplemented with glutamine, 10% heat inactivated Fetal Bovine Serum, 50 IU/mL Penicillin, 50 μg/mL Streptomycin, 10 mM HEPES and 0.05 mM β-mercaptoethanol. Cells were treated with 1% Echinaforce® tincture versus ethanol solvent control. Each treatment condition consisted of six biological replicates.

### Genome-wide gene expression analysis

#### Sample preparation and microarray processing

THP1 cells were treated for 48 h with 1% Echinaforce® or ethanol solvent control. RNA was isolated using the RNeasy mini kit (Qiagen) according to manufacturer’s instructions. RNA concentration and purity was measured using the Nanodrop 1000 spectrophotometer (ThermoFischer, CA, USA). RNA integrity of each sample was checked using using the Experion Automated Electrophoresis System (Bio-Rad, MO, USA). Total RNA (500 ng) was amplified using the Illumina TotalPrep RNA Amplification kit (Life Technologies, Carlsbad, CA, USA). Briefly, RNA was reverse transcribed using T7 oligo (dT) primers, after which biotinylated complementary or anti-sense RNA (cRNA) was synthesized through an in vitro transcription reaction. Then, 750 ng of amplified cRNA was hybridized to a HumanHT12 beadchip array (Illumina, San Diego, CA, USA) and further incubated for 18 h at 58 °C in a hybridization oven under continuous rocking. After several consecutive washing steps, bead intensities were read on an Illumina iScan. Microarray data and raw gene expression intensities were preprocessed and analyzed using the beadarray R package [30]. Intensities were quantile normalized and log_2_ transformed. Raw and normalized array data were uploaded to the Gene Expression Omnibus (GEO) database and have accession number: GSE117904. Probes with a P-detection value higher than 0.05 in at least six samples were removed. Also, probes annotated as “bad” and “no match” as described before [31] were not kept for further analysis. Differentially gene expression was performed using the limma R package [32]. *P*-values were corrected for multiple testing using the method of Benjamini and Hochberg. Probes with a log2 fold change higher than 0.4 and an adjusted *p*-value less than 0.05 were defined as significant and kept for further analysis [33]. The probes were annotated with gene information using the illuminaHumanv4.db annotation dataset [34]. The gene IDs of the significant Illumina expression probes were uploaded into the IPA software (Ingenuity® Systems, www.ingenuity.com, Redwood City, CA, USA) to find enriched biological pathways, diseases and networks [35]. Fischer ‘s exact test was used to calculate a *p*-value determining the probability that each biological function and/or disease assigned to that data set is due to chance alone. Metascape systems biology freeware (https://metascape.org/) was used for correlating the transcriptomic profile data [36]. For each given gene list, pathway and process enrichment analysis has been carried out with the following ontology sources: KEGG Pathway, GO Biological Processes, Reactome Gene Sets, Canonical Pathways, CORUM, TRRUST, DisGeNET, PaGenBase, Transcription Factor Targets, WikiPathways, PANTHER Pathway and COVID. All genes in the genome have been used as the enrichment background. Terms with a *p*-value < 0.01, a minimum count of 3, and an enrichment factor > 1.5 (the enrichment factor is the ratio between the observed counts and the counts expected by chance) are collected and grouped into clusters based on their membership similarities. More specifically, *p*-values are calculated based on the accumulative hypergeometric distribution, and q-values are calculated using the Banjamini-Hochberg procedure to account for multiple testings. Kappa scores are used as the similarity metric when performing hierachical clustering on the enriched terms, and sub-trees with a similarity of > 0.3 are considered a cluster. The most statistically significant term within a cluster is chosen to represent the cluster. Heatmaps show Metascape enrichment analysis of all statistically enriched ontology terms (GO/KEGG terms, canonical pathways, hall mark gene sets). Accumulative hypergeometric *p*-values and enrichment factors are calculated and used for filtering. Remaining significant terms are then hierarchically clustered into a tree dendrogram based on Kappa-statistical similarities among their gene memberships. The term with the best *p*-value are selected within each cluster as a representative term to be displayed in a hierarchical tree dendrogram. The heatmap cells are colored by their *p*-values (see color legend). Along the same line, Metascape enrichment analysis of all statistically enriched TF-target interaction networks is dermined by the TRRUST database [37]. Protein-protein interactions (PPI) among all input gene lists are extracted from PPI data source to form a PPI network (interactome). GO enrichment analysis is applied to the network to assign biological “meanings” of sub-protein networks. GO enrichment analysis is applied to each MCODE network to assign “meanings” to the network component, where top three best *p*-value terms were retained. MCODE components were identified from the merged network. Each MCODE network is assigned a unique color. For each given gene list, protein-protein interaction enrichment analysis has been carried out with the following databases: STRING, BioGrid, OmniPath, InWeb_IM. Only physical interactions in STRING (physical score > 0.132) and BioGrid are used. The resultant network contains the subset of proteins that form physical interactions with at least one other member in the list. If the network contains between 3 and 500 proteins, the Molecular Complex Detection (MCODE) algorithm [38] has been applied to identify densely connected network components. The MCODE networks identified for individual gene lists have been gathered and are summarized in the MCODE subnetwork figure. Pathway and process enrichment analysis has been applied to each MCODE component independently, and the three best-scoring terms by *p*-value have been retained as the functional description of the corresponding MCODE components. Coronascape is a Metascape data hub including public available COVID-19 research related omics data sets. It includes more than 200 processed gene lists for SARS-CoV-2 retrieved from more than 20 published studies. These gene lists were generated using several omics technologies, including transcriptome (RNA-Seq and scRNASeq), proteome, phosphoproteome, ubiquitome, and interactome, providing a comprehensive picture of SARS-CoV-2 infection in various host cell and tissue types.

### Quantitative realtime PCR

To validate microarray data, THP1 cells were treated with 1% Echinaforce**®** or Solvent for the indicated time-points (3, 6, 12, 24 and 48 h) in three independent experiments. The effect of JAK1 inhibition was determined by treating the cells with 1 μM JAK1 inhibitor Filgotinib (GLPG0634, Selleckchem) for 30 min before adding Echinaforce**®**. Total RNA was isolated using the RNeasy mini kit (Qiagen, Hilden, Germany) including a DNAse treatment step as suggested by the manufacturer. Then 750 ng RNA was reverse transcribed into cDNA using oligo dT (Invitrogen), M-MLV reverse transcriptase (Promega, Wisconsin USA), 2.5 mM dNTPs and RNaseOUT (Invitrogen). Samples were incubated on 42 °C for 60 min and 75 °C for 15 min. For the HERV genes, cDNA synthesis was performed using random primers (Invitrogen) and incubation of the samples at 37 °C for 60 min and 75 °C for 15 min. qPCR was performed using the GoTaq qPCR Master Mix (Promega, Wisconsin USA) on a StepOnePlus Real-Time PCR machine (Applied Biosystems). Following primers were used: MX1 forward primer 5′-GTTTCCGAAGTGGACATCGCA-3′, MX1 reverse primer 5′-CTGCACAGGTTGTTCTCAGC-3′ (NM_001144925), IFITM1 forward primer 5′-CCAAGGTCCACCGTGATTAAC-3′, IFITM1 reverse primer 5′-ACCAGTTCAAGAAGAGGGTGTT-3′ (NM_003641), STAT1 forward primer 5′- CCATCCTTTGGTACAACATGC-3′, STAT1 reverse primer 5′-TGCACATGGTGGAGTCAGG-3′ (NM_007315), IL8 forward primer 5′-GCTCTCTTGGCAGCCTTCCTGA-3′, IL8 reverse primer 5′-ACAATAATTTCTGTGTTGGCGC-3′ (NM_000584), CXCL10 forward primer 5′-GAAAGCAGTTAGCAAGGAAAGGT-3′, CXLC10 reverse primer 5′-GACATATACTCCATGTAGGGAAGTGA-3′ (NM_001565), ACTB forward primer 5′-CTGGAACGGTGAAGGTGACA-3′, and ACTB reverse primer 5′- AAGGGACTTCCTGTAACAATGCA-3′ (NM_001101). Primer sequences for HERVs were derived from [39]. Each sample was ran in triplicate and the median Ct-values between each replicate group was selected. Ct-values were normalized using ACTB housekeeping gene. The ddCt-values or log fold changes (logFC) were calculated using the solvent control as reference sample. A paired t-test t-test was used to determine the significance of the differences between Echinaforce**®** and solvent expression levels.

### Kinase activity profiling

#### Sample preparation

THP1 cells were treated with 1% Echinaforce® or ethanol solvent control for 15 min. Cell lysates were prepared according to manufacturer’s instructions. In short, cells were washed twice with cold 1X PBS and lysed with lysis buffer (1:100 dilution of Halt Phosphatase Inhibitor Cocktail and Halt Protease Inhibitor Cocktail EDTA free in M-PER Mammalian Extraction Buffer (ThermoFisher Scientific™, Rockford, USA) at a ratio of 100 μl buffer per 1 × 10^6^ cells. Lysates were then incubated on ice for 15 min and centrifuged for 15 min at 16000 x g at 4 °C. Protein concentration was quantified using the Pierce BCA Protein Assay Kit (ThermoFisher Scientific™, Rockford, USA).

#### Serine/threonine kinases (STK) and tyrosine kinase (PTK) pamgene assay and data analysis

Kinase activity profiling was performed PamChip® preprocessing and kinase activity profiling was performed according to manufacturer’s instructions (PamGene International BV, ‘s-Hertogenbosch, The Netherlands). The first part of the protocol consisted in the blocking of the arrays with 2% BSA followed by several washing steps. Then 0.5 μg for STK and 5 μg for PTK assays together with the correspondent reaction mixes (purchased from the Pamgene) were loaded onto the arrays and incubated in the microarray system PamStation® 12 instrument (PamGene International, Den Bosch, The Netherlands). In this step, the ATP contained in the mix leads to the activation of the kinases in the lysate which will result in the phosphorylation of the peptides on the array. Peptide phosphorylation intensities are then detected with the primary STK antibody mix and FITC-labeled antibody for STK assay and with the FITC-labelled PTK antibody (PTK assay). Images are then taken by the CCD camera in the PamStation®12 and processed by the Bionavigator software. Peptide intensities data were log_2_ transformed and differences in phosphorylation between Echinaforce® treated and control cultures were determined by using an univariate student t-test analysis corrected for multiple testing using the Benjamini and Hochberg method [33].

To identify potentially activated or inhibited kinases we used the STK or PTK Upstream Kinase analysis PamApp from the Bionavigator Software. The analysis is based on “in silico predictions” for the upstream kinases of phosphorylation sites in the human proteome that are retrieved from the phosphoNET database [40]. In short, a prediction algorithm is derived from known interactions between kinases and phosphorylation sites. The prediction algorithm is then used to predict the strength of undocumented interactions. The Bionavigator application uses PhosphoNet database to map putative kinases upstream of the phospho-peptides (a kinase can have multiple possible phosphosites, and a single site can be phosphorylated by different kinases). For each set of peptides mapped to a specific kinase, a “difference statistics” is calculated (=normalized kinase statistics) using following formula: $\tau =\frac{1}{n}{\sum}_{i=1}^n\frac{{\overline{p}}_{i1}-{\overline{p}}_{i2}}{\sqrt{s_{i1}^2+{s}_{i2}^2}}$ with ${\overline{p}}_{ij}$ and ${\overline{s}}_{ij}$ as the sample mean and variance of the intensity of peptide i in group j, respectively, whereas n is the number of peptides linked with a specific kinase. A positive kinase statistic means that the kinase is activated, while a negative statistic means the kinase is inactivated compared to the control group. The kinases are subsequently ranked based on a specificity and significance score which are calculated using permutation of the peptides and samples, respectively. Following formula is used: $Q=-{\log}_{10}\left(\max \left(\frac{m}{M},\frac{1}{M}\right)\right)$, where m is the number of times out of M permutations that |τ_p_| > |τ|, where τ_p_ is the value of the difference statistic obtained after permutation of the samples or peptides. The significance score represents the magnitude of the change represented by the normalized kinase statistic. The specificity score represents the specificity of the of normalized kinase statistic in terms of the set of peptides used for the corresponding kinase. The higher the score the less likely it is that the observed normalized kinase statistics could have been obtained using a random set of peptides from the data set. The sum of the significance and specificity score is used to rank the kinases [41].

### Genome-wide DNA methylation analysis

#### Sample preparation

THP1 cells were cultured for 48 h with 1% Echinaforce® or ethanol solvent control. Corresponding cellular genomic DNA was isolated using the DNeasy Blood & Tissue kit (Qiagen, Hilden, Germany) according to manufacturer’s instructions. DNA concentration and purity was measured using the Nanodrop 100 spectrophotomer and 1 μg of DNA was used for bisulfite conversion using the EZ DNA methylation Kit of Zymo Research according to manufacturer’s instructions. Successful bisulfite conversion was checked using a methylation-specific PCR in a region of the SALL3 gene (see [42] for primer sequences).

#### EPIC DNA methylation array

The Infinium HumanMethylationEPIC BeadChip array (Illumina, San Diego, CA, USA) was used to measure genome-wide DNA methylation. Four μL of bisulfite-converted DNA from each sample was amplified, fragmented, precipitated, resuspended and subsequently hybridized onto the BeadChips. After overnight incubation of the BeadChips, unhybridized fragments were washed away, while hybridized fragments were extended using fluorescent nucleotide bases. Finally, the BeadChips were scanned using the Illumina iScan system to obtain raw methylation intensities of each probe.

#### EPIC DNA methylation data preprocessing and analysis

The R package RnBeads was used to preprocess the Illumina 450 K methylation data [43]. CpG-probes were filtered before normalization based on following criteria: probes containing a SNP within 3 bp of the analyzed CpG site, bad quality probes based on an iterative greedycut algorithm with a detection *p*-value threshold of 0.01, and probes with missing values in at least one sample. After filtering these CpG-probes, methylation values were within-array normalized using the beta mixture quantile dilation (BMIQ) method [44]. Another filtering step was performed after normalization based on following criteria: probes measuring methylation not at CpG sites (CC, CAG, CAH, …) and probes on sex chromosomes.

The methylation beta-values were transformed to M-values (M = log_2_(β/(1-β))) prior to further analyses. The moderated t-test incorporated in the limma R package [32] was used to calculate the statistics and *p*-values of the methylation differences between Echinaforce®- and solvent-treated samples. Significant differentially methylated probes (DMPs) were selected based on a false discovery rate (FDR) < 0.1 and a difference in beta-value of at least 0.05. The DMPs were annotated with gene information using the IlluminaHumanMethylationEPICmanifest R package [45]. Further gene information was retrieved from the UCSC genome browser (human hg19). Enrichment of genomic regions was calculated using the Fisher’s exact test. Pathway analysis of the genes harboring a DMP was performed using the Ingenuity Pathway Analysis (IPA) software. Raw and normalized array data were uploaded to the Gene Expression Omnibus (GEO) database and have accession number: GSE117904.

### Protein expression of MX1, STAT1 and IFITM1 proteins using western blotting

Protein expression levels of MX1, STAT1 and IFTIM1 were determined in THP1 cells treated with 1% Echinaforce® or ethanol solvent control for 48 h, as explained before. Then, cells were washed and incubated 15 min on ice in lysis buffer containing: 150 mM NaCl, 1 mM EGTA, 1 mM EDTA, 1 mM ß-glycerolphosphate, 1% Triton X-100 (w/v), 20 mM Tris HCl, pH = 7.5 and proteinase inhibitor (Complete™, EDTA-free Protease Inhibitor Cocktail, Sigma-Aldrich, USA) plus PhosphataseArrest™ Phosphatase Inhibitor Cocktail (phosphataseArrest™, G-Biosciences, USA). Cells were subsequently centrifuged for 15 min at 200 g at 4 °C and supernatant containing the soluble proteins were stored at − 20 °C until use. Protein lysates (20 μg) were mixed with 5X sample buffer (5% SDS, 20% glycerol, 0.2% bromophenol-blue, 250 mM DTT, 65 mM Tris HCl) all purchased from Sigma Aldrich (Missouri, USA), heated for 5 min at 95 °C and loaded in a 12% SDS-PAGE gel. Proteins contained in the homogenates were separated during 30 min at 60-70 V and 1 h at a constant voltage of 130 V. Further, 10 μl of BenchMark™ Pre-Stained Protein Standard (Life Technologies, CA, USA) was also loaded next to the samples. After separation proteins ttransferred onto a Nitrocellulose Membrane (BioRad, CA, USA) during 2 h at 45 V. Non-specific binding sites were blocked by incubating the membranes with blocking buffer (0.05% Tween 20, 1x TBS, 5% BSA) for 1 h at room temperature. The membrane was then incubated with the primary antibodies: MX1 (D3W7I) Rabbit mAb #37849, IFITM1 Antibody Rabbit pAb #13126 and the STAT1 (42H3) Rabbit mAb #9175 (all purchased from Cell Signaling Technology, Massachusetts, USA) or rabbit polyclonal Anti-GAPDH antibody (ab9485, Abcam, Cambridge, UK) overnight at 4 °C. After membranes were washed, they were incubated with (1:10000) Donkey anti-Rabbit IgG (H + L) Secondary Antibody-HRP (Thermo Fisher Scientific, Massachusetts, USA) for 1 h at room temperature. Chemiluminiscence detection was performed using the ECL detection kit (Pierce™ ECL Western Blotting Substrate (Thermo Fisher Scientific, Massachusetts, USA) in a ChemiDoc MP system (BioRad, CA, USA).

### Assessment of IFNα2, IFNβ IFNγ, CXCL8 (IL8) and CXCL10 levels

Cell culture supernatants were collected after 3, 6, 12, 24 and 48 h and assayed for chemokines CXCL10 and IL8 by means of an enzyme-linked immunosorbent assay (ELISA) purchased from Invitrogen (CA, USA) following manufacturer’s instructions. The assays have a detection limit of 2 pg/ml for CXCL10 and 5 pg/mL for IL-8. Similarly, protein concentrations of IFNα2, IFNβ and IFNγ were measured in the same culture supernatants using the highly sensitive U-PLEX Biomarker Group 1 (hu) Assay (Meso Scale Diagnostics, Maryland, USA) following manufacturer’s instructions. The U-PLEX assays have a detection limit of 4.0 pg/ml, 3.1 pg/mL and 1.7 pg/mL respectively for IFNα2, IFNβ and IFNγ.

## Results

### Echinaforce® treatment triggers tonic IFN regulation of innate immunity signaling pathways

**Fig. 1:**
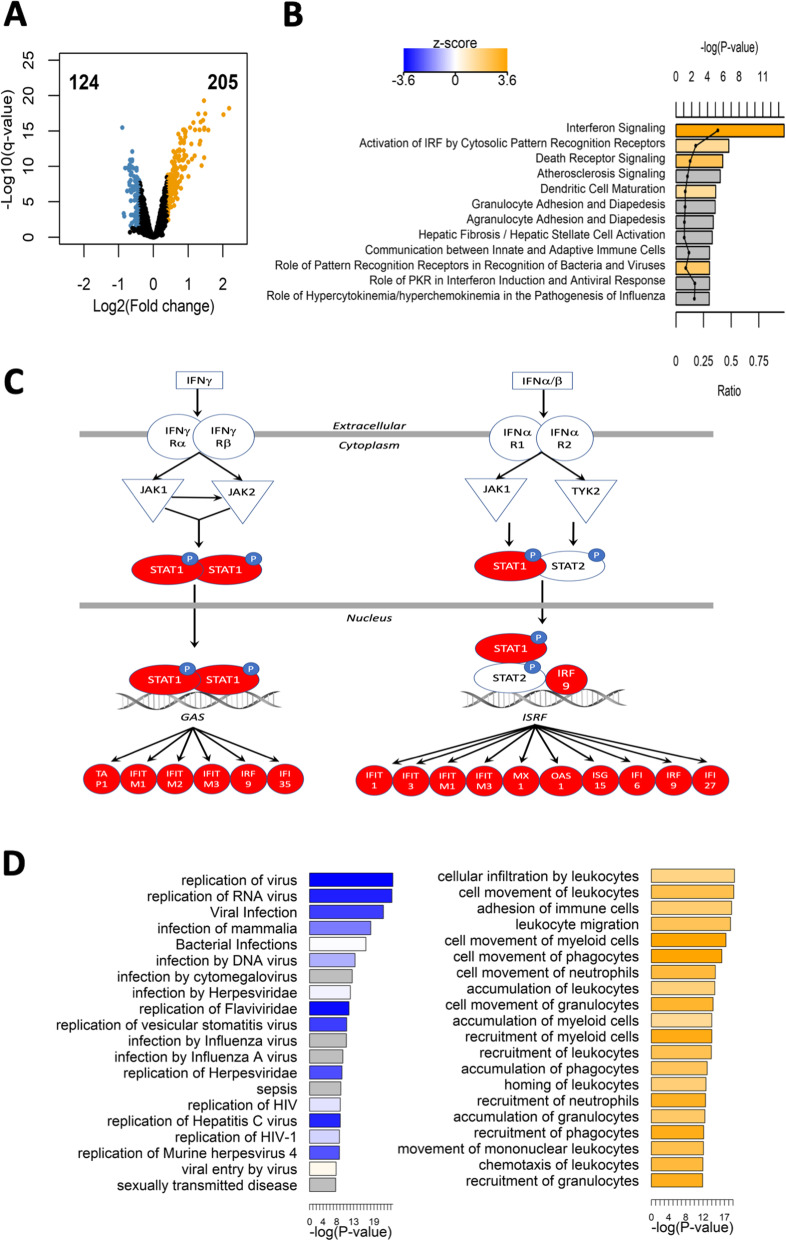
Echinaforce® induced gene expression activates innate immunity pathways **a** Volcano plot showing the upregulated genes (orange color, number of probes: 205), and downregulated genes (blue color, number of probes: 124) upon treatment of THP1 cells for 48 h with Echinaforce® tincture (1%). **b** Top enriched IPA canonical pathways. Bars are colored by activation z-score. **c** IPA interferon signaling pathway with Echinaforce®-induced upregulated genes colored in red and green, respectively. **d** Top enriched IPA infectious diseases and IPA immune trafficking disease and biological function. Bar charts are colored by activation z-score

Widespread gene expression changes in monocyte THP1 cells were detected upon 48 h 1% Echinaforce® treatment. Based on significance criteria of FDR < 0.05 and absolute log_2_ fold change > 0.4, Echinaforce® induced modest upregulation of 205 expression probes (173 genes) while 124 probes (99 genes) were downregulated compared with the ethanol treated solvent controls (Fig. 1**a and Supplementary Table** **1**). In contrast to pharmacological drugs (for example glucocorticoids (GC)) which can trigger drastic expression changes of GC-responsive genes (typically, log_2_ fold > 1), many bioactive phytochemicals rather induce moderate transcriptional changes (typically log_2_ fold > 0,4) of multiple genes converging on the same pathway [46–48]. Genes differentially expressed (DEG) by Echinaforce® treatment were enriched for IPA canonical pathways related to innate immune responses including interferon signaling, interferon regulatory factor (IRF) activation and the role of pattern recognition receptors, among others (Fig. 1**b-c and Supplementary Table** **2**). Interestingly, most of these pathways were predicted to be activated, as can be seen from the highly positive activation z-scores. Interferon (IFN)α/β and IFNγ both induce IFN-stimulated gene (ISG) expression through Janus kinase (JAK)-dependent phosphorylation of signal transducer and activator of transcription factors (STAT)1 and STAT2 [49–54]. In line with the latter reports, we could observe transcriptional activation of various antiviral gatekeepers and interferon inducible proteins (i.e. MX1, IFI6/27/35/44, IFITM1/2/3, IFIT1/2/3, ISG15/20, IRF7/9), including various STAT1 target genes (Fig. 1**c, Supplementary Table** **1****,** **2**). Logically, pathways related to viral infection and replication were predicted to be inhibited (activation z-score < − 2). Also pathways involving cellular movement, migration, recruitment and chemotaxis were predicted to be activated (activation z-scores > 2) (Fig. 1**d**). Aside from ISGs, transcription of various chemokines and receptors (CXCL10, CXCL8, CCL2, CCL5, and CXCR4) were also increased. In full accordance, recruitment and adhesion of immune cells, infection and immune related processes were found top ranked enriched diseases and biological functions in IPA analysis (**Supplementary Table** **3****)**.

**Fig. 2:**
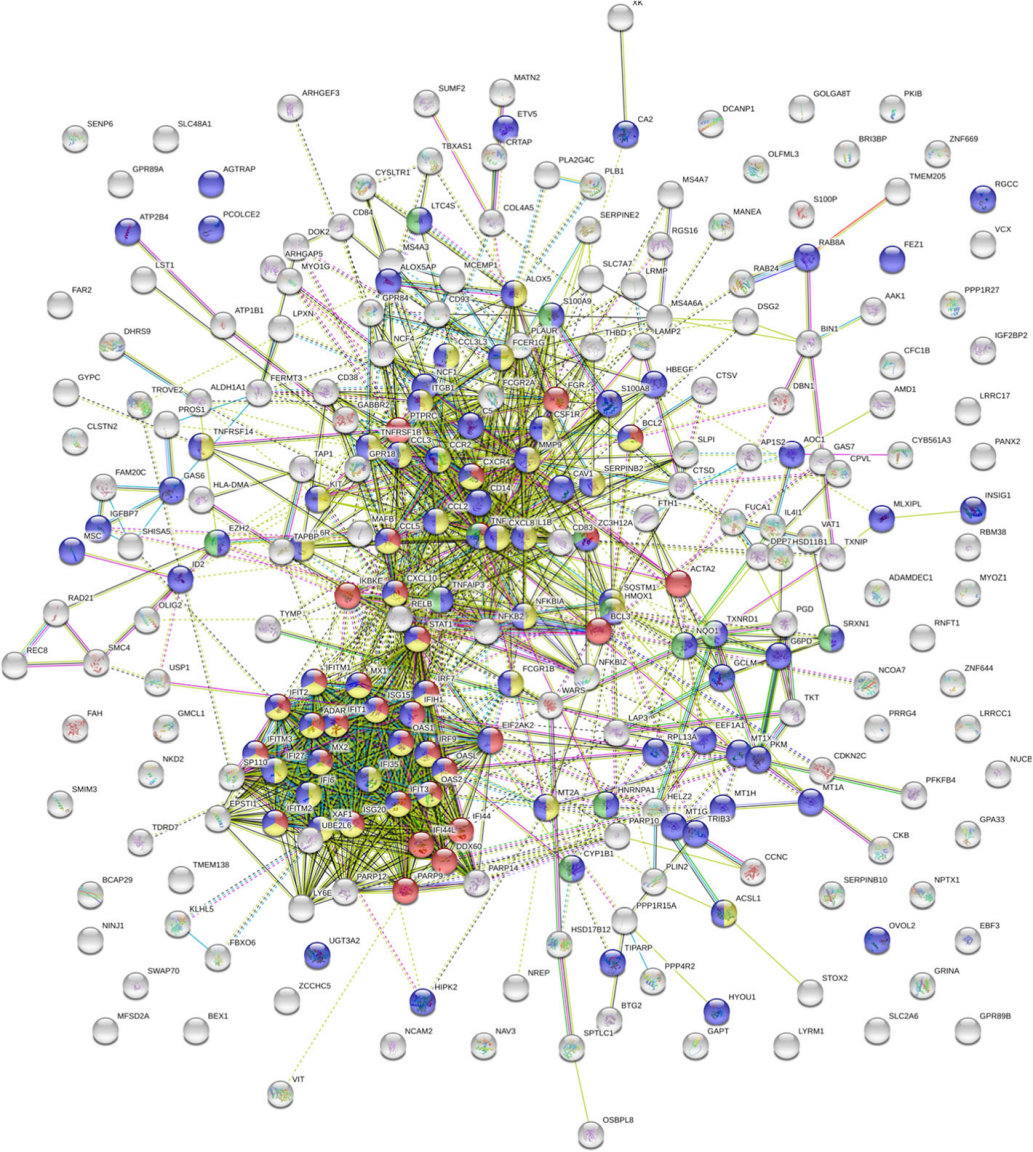
Protein-protein-interaction network analysis of Echinaforce® treatment responsive genes. STRING based protein-protein-interaction network analysis of differentially expressed genes of THP1 cells treated for 48 h with Echinaforce® tincture (1%) shows a strong network overlap of the cellular response to a chemical stimulus (FDR 2,91 E-18) (blue colored dots-pies), cellular defense to virus (FDR 5,54 E-19) (red colored dots-pies) and innate immune cytokine response (FDR 2,23 E-18) (yellow colored dots-pies) (see also, supplementary Table 4)

Complementary to IPA analysis, protein-protein-interaction enrichment analysis of DEGs by STRING [55] and Metascape [56] algorithms was performed. This revealed strong enrichment of protein-protein interactions responding to a chemical stimulus, which triggers a defensive antiviral innate immune response involving IFN, TLR, NOD, RIG, cytokine, chemokine and NFκB signaling pathways (Fig. 2, **Supplementary Table** **4**). More particularly, Metascape MCODE analysis identified 3 interconnected subnetworks in the antiviral cytokine response: cellular response to interferon, regulation of leukocyte chemotaxis and (mitochondrial) metabolism (**Supplementary Table** **4**).

**Fig. 3:**
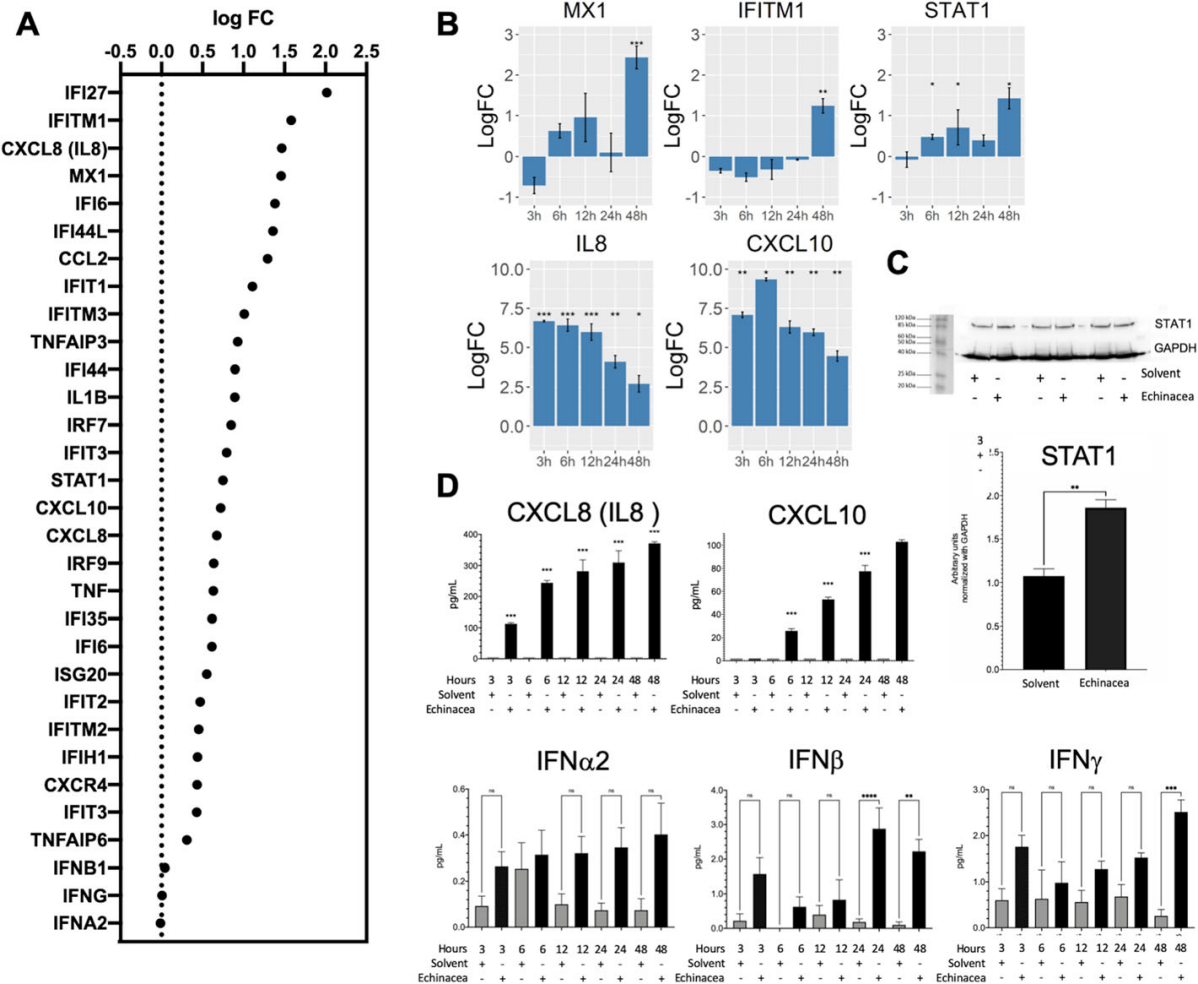
Induction of innate immune response by Echinaforce®. A) transcriptome gene expression changes IFN, innate immunity, chemokine, cytokine genes (logFC) B) transcription levels of MX1, IFITM1, STAT1, CXCL8(IL8) and CXCL10 genes at different time points, the bars represent the mean logFC values + − SD compared to the solvent control. *: *P* ≤ 0.05, ** *P*: ≤ 0.01, *** *P*: ≤ 0.001 and **** *P*: ≤ 0.0001. C) Blots showing protein levels of STAT1 and GAPDH (as reference protein) in 20 μg protein of cell lysates after 48 h stimulation with solvent (Ethanol) or Echinaforce®; Bars graph represents the density of each blot band for STAT-1 relative to the band density of GAPDH (reference protein). Band intensities were calculated using imageJ software. Statistical differences between solvent and Echinacea treated samples were assayed using a paired t-test where *p* value < 0.05 was considered statistically significant. (***) means p value < 0.01, (*): *P* ≤ 0.05, (**): *P* ≤ 0.01, (***): *P* ≤ 0.001 and (****): *P* ≤ 0.0001. D) Expression levels of IL8, CXCL10, IFNα2, IFNβ, IFNγ chemokines assayed by ELISA and MSD-U-Plex immunoassays in supernatants collected after Echinaforce® and solvent (Ethanol) stimulation. (***) means p value < 0.01, (*): *P* ≤ 0.05, (**): *P* ≤ 0.01, (***): *P* ≤ 0.001 and (****): *P* ≤ 0.0001, *p*-values after a paired t-test where *p* value < 0.05 was considered statistically significant

Next, different gene members of the IFN and chemotaxis innate immune signaling pathway, responsive to Echinaforce® treatment in THP1 cells (Fig. 3a) were selected for further evaluation of time dependent expression changes: STAT1, MX1, IFITM1, IFNα2, IFNβ, IFNγ, CXCL8 and CXCL10 mRNA and/or protein levels were measured in THP1 monocytes after 3 to 48 h Echinaforce® treatment by means of qPCR, ELISA, multiplex MSD U-PLEX® immunoassay and/or Western immunoblotting assays. Induction of STAT1 and the interferon-stimulated genes MX1 and IFITM1 expression could clearly be confirmed, with maximal mRNA transcription levels observed after 48 h treatment (Fig. 3**b**). Corresponding changes in STAT1 protein expression levels could also be verified by Western analysis (Fig. 3**c**), whereas antibodies failed to detect significant amounts of MX1 and IFITM1 protein (data not shown). Whether MX1 and IFITM1 protein expression has high turnover rates resulting in low protein expression levels needs further investigation [57, 58]. For the chemokines IL8 and CXCL10, persistent gene induction could be observed upon Echinaforce® treatment until 48 h, with peak transcription levels after 3 h (Fig. 3**b**). Accordingly, time dependent accumulation of both chemokines in the cell culture supernatants could be detected in ELISA (Fig. 3**d**). Finally, in line with background mRNA transcription levels, multiplex immunoassay detection of supernatant levels of IFNα2, IFNβ, IFNγ protein only showed low expression levels, which weakly increase after 48 h Echinaforce® treatment Fig. 3**e)**. However, in contrast to high expression levels of IFN upon acute viral infection, very weak expression levels of IFN in absence of infection also exert profound immunological effects, in part through “tonic” homeostatic modulation of various signaling intermediates which regulate diverse cytokines to train immunity [59–61].

### Echinaforce® treatment activates IFN and antiviral innate immune response which is suppressed in severe SARS-CoV-2 patients

**Fig. 4:**
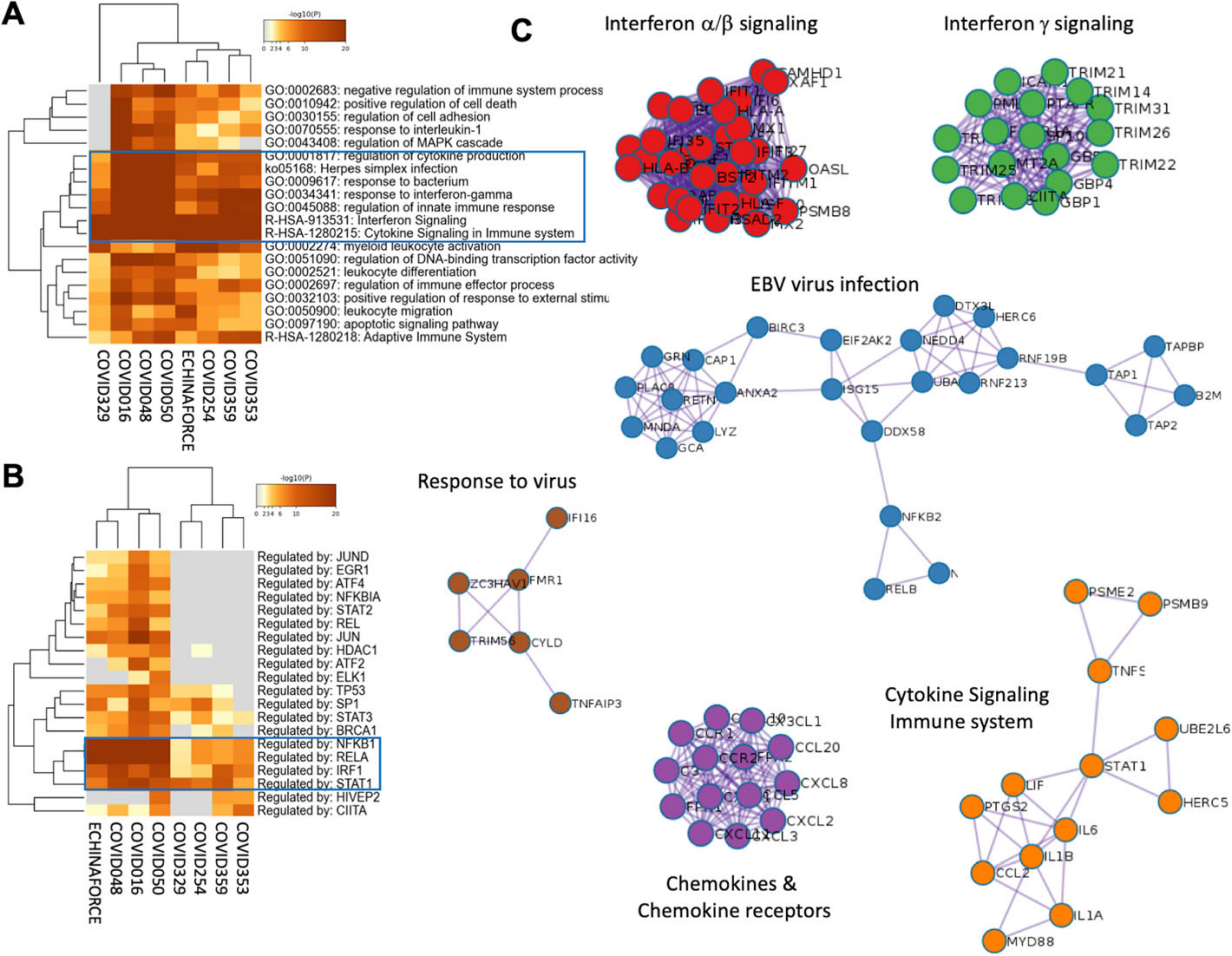
Systems level metascape analysis of transcriptome profiles of Echinaforce treated THP1 monocytes and blood PBMC samples of SARS-CoV2 patients. **a** Metascape enrichment analysis of statistically enriched ontology terms (GO/KEGG terms, canonical pathways, hall mark gene sets). **b** Metascape enrichment analysis of all statistically enriched TF-target interaction networks **c** GO enrichment analysis of all protein-protein interaction networks to assign biological function to each MCODE sub-protein-networks

Coupled to Metascape analysis [36], the Coronascape database (https://metascape.org/COVID) provides quick access to numerous published COVID-19 omics data sets, and a comprehensive system level data analysis toolkit for data mining. Remarkably, upon comparison of our Echinaforce® responsive gene signature in THP1 monocytes with public available datasets of gene expression profiles of SARS-CoV2 patients, we observed a very strong overlap in enriched pathways (*P*-value 10^− 48^–10^− 61^) related to IFN, cytokine and innate immune signaling in patients with mild to severe symptoms [62–69] (Fig. 4**a**). Of special note, whereas Echinaforce® treatment was found to promote innate immunity via multiple IFN stimulated genes (ISG), i.e. pattern recognition receptor genes and chemokines (our results), severe SARS-Cov2 patients typically suffer from a strongly impaired interferon (IFN) type I response and weak innate antiviral defense (ISGs), associated with a persistent blood viral load and an exacerbated inflammatory response [64–72]. Furthermore, Metascape TRRUST analysis [37] of all statistically enriched TF binding motifs in differentially expressed genes in severe covid patients, which can be modulated by Echinaforce treatment identified key roles for NFκB, STAT and IRF family transcription factors (Fig. 4**b**). Finally, Metascape Protein-protein interaction analysis of Echinaforce regulated protein networks identified multiple antiviral IFN and immune signaling networks disturbed in severe SARS-CoV2 patients (Fig. 4**c, Supplementary Table** **4**), including an EBV specific virus infection protein network. Remarkably, EBV reactivation and increased EBV DNA load have recently been reported in severe SARS-CoV2 patients with impaired lymphocyte subpopulation counts [73].

### Echinaforce*®* treatment activates JAK1, NFκB and MAPK kinases

**Fig. 5:**
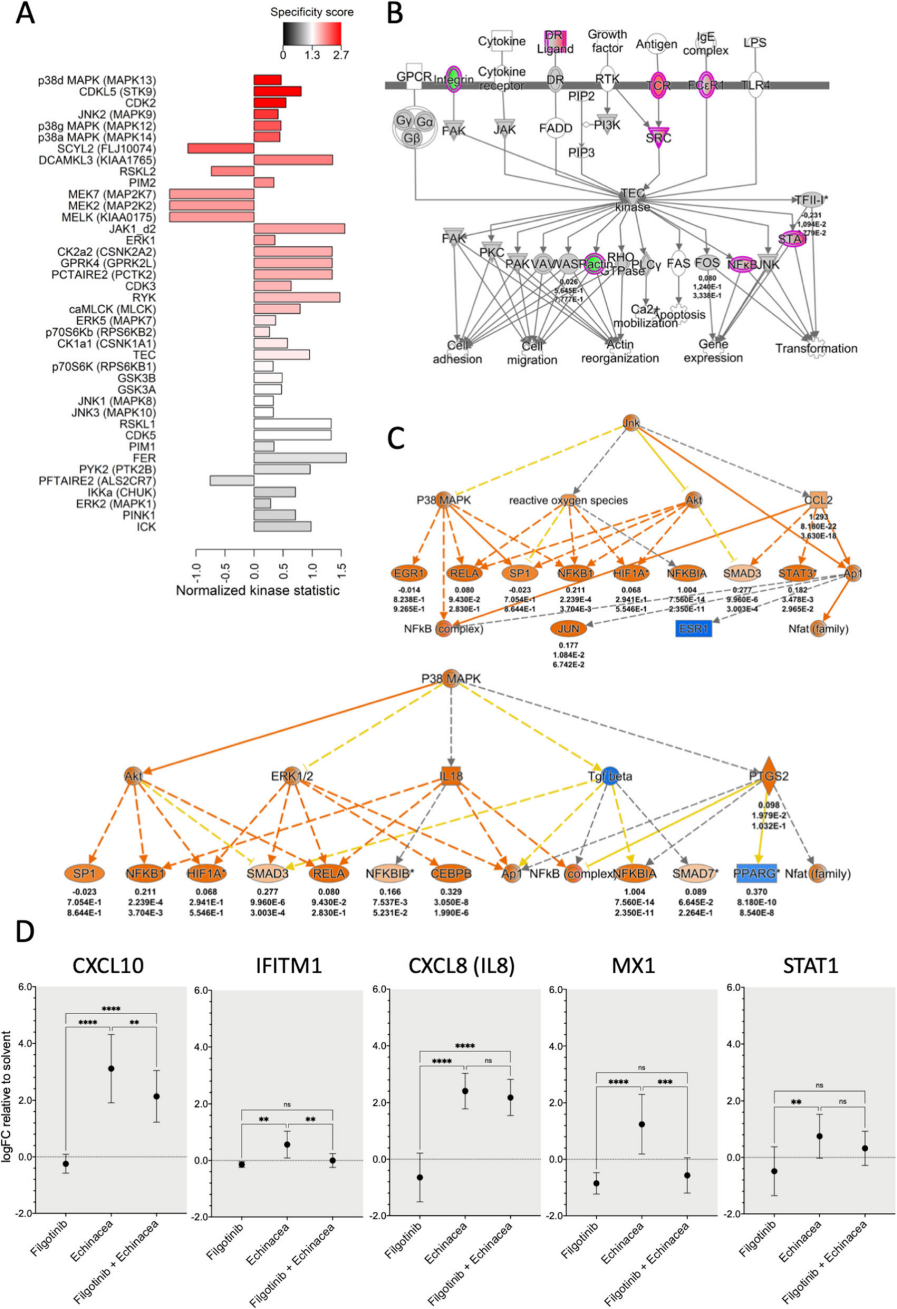
Activation of JAK1 and MAPK kinases by Echinaforce®. **a** Kinome activity profiling on THP1 cell lysates, following 15 min treatment with Echinaforce® tincture (1%). Showing predicted upstream kinases. Bars are colored by specificity score with red meaning the highest score. The direction of the bars represents the normalized kinase statistics. A positive kinase statistic means a higher activity in Echinaforce® treated samples. **b** TEC signaling pathway as predicted by IPA software showing the up- and down-regulated genes (colored in red and green, respectively), after Echinaforce® treatment. Numbers under genes names represent (from up to down): the log fold changes, *p*-values and adjusted *p*-values after a paired t-test comparing gene expression in cells stimulated with Echinaforce® and solvent (control). **c**) IPA-enriched P38 MAPK and JNK pathways upstream regulators. Genes colored in orange are predicted to be activated, while genes colored in blue are predicted to be inhibited. Numbers under gene names represent (from up to down): the log fold changes, p-values and adjusted *p*-values after a paired t-test comparing gene expression in cells stimulated with Echinaforce® and solvent (control). **d** Effect of JAK1 inhibition on transcript expression of interferon pathway related genes. THP1 cells were either treated during 48 h with the JAK1 inhibitor Filgotinib alone or in combination with Echinaforce® (*n* = 7). Mean expression LogFC change relative to solvent control is represented together with 95% confidence interval. *: *P* ≤ 0.05, ** *P*: ≤ 0.01, *** *P*: ≤ 0.001

To identify most important upstream kinase pathways responsible for gene expression changes in THP1 monocytes following Echinaforce® treatment, we performed a Pamchip kinome activity profiling assay [41]. This peptide array approach allows characterization of cellular serine/threonine or tyrosine kinome activity profiles following on chip in vitro kinase reaction of 144 conserved kinase consensus peptide motifs in presence of THP1 monocyte lysates left untreated or following Echinaforce® treatment [74–77]. Using the upstream kinase prediction tool of the Bionavigator PamGene software, the qualitative and quantitative changes in phosphopeptide chip intensities upon Echinaforce® treatment were translated into a pattern of activated or inhibited upstream kinases (Fig. 5**a and Supplementary Table** **5**). In agreement with the transcriptional activation of the IFN signaling pathway described above (Fig. 1**c**), Pamchip kinome profiling [41] revealed activation of the JAK1 kinase which is important in the phosphorylation of STAT kinases and subsequently downstream regulation of IFN-stimulated genes. Furthermore, in line with pathway analysis of transcriptome data, we also identified activation of the tyrosine kinase TEC (Fig. 5**b**) (**Supplementary Table** **2****,** **3****,** **4**). Surprisingly, our analysis did not detect significant activity changes of early IFN kinases TBK1 and IKK [78].

Besides, we also identified various Echinaforce® activated kinases belonging to the MAPK superfamily of kinases: p38 MAPK (MAPK11, − 12, − 13, and − 14), JNK (MAPK8, − 9 and − 10) and ERK1 (Fig. 5**c**). This upstream regulators are also predicted by IPA to control various canonical pathways, including pattern recognition receptors in recognition of bacteria and viruses, activation of IRF by cytosolic pattern recognition receptors and role of MAPK signaling in the pathogenesis of influenza among others.

To further verify crucial involvement of JAK kinase activation in downstream gene expression effects upon Echinaforce® treatment, we compared THP1 gene expression changes following Echinaforce® treatment in presence or absence of the pharmacological JAK1 inhibitor filgotinib. We found that filgotinib significantly suppresses the Echinaforce® responsive genes MX1 and IFITM1, whereas STAT1, CXCL10 and IL8 gene expression were less significantly suppressed (Fig. 5**d**). Altogether, experiments with the JAK1 inhibitor filgotinib strenghten our transcriptome and kinome data analysis, pointing to JAK1-specific regulation of downstream gene expression changes in response to Echinaforce® treatment.

### Echinaforce® treatment elicits epigenetic changes in innate immunity gene pathways

**Fig. 6:**
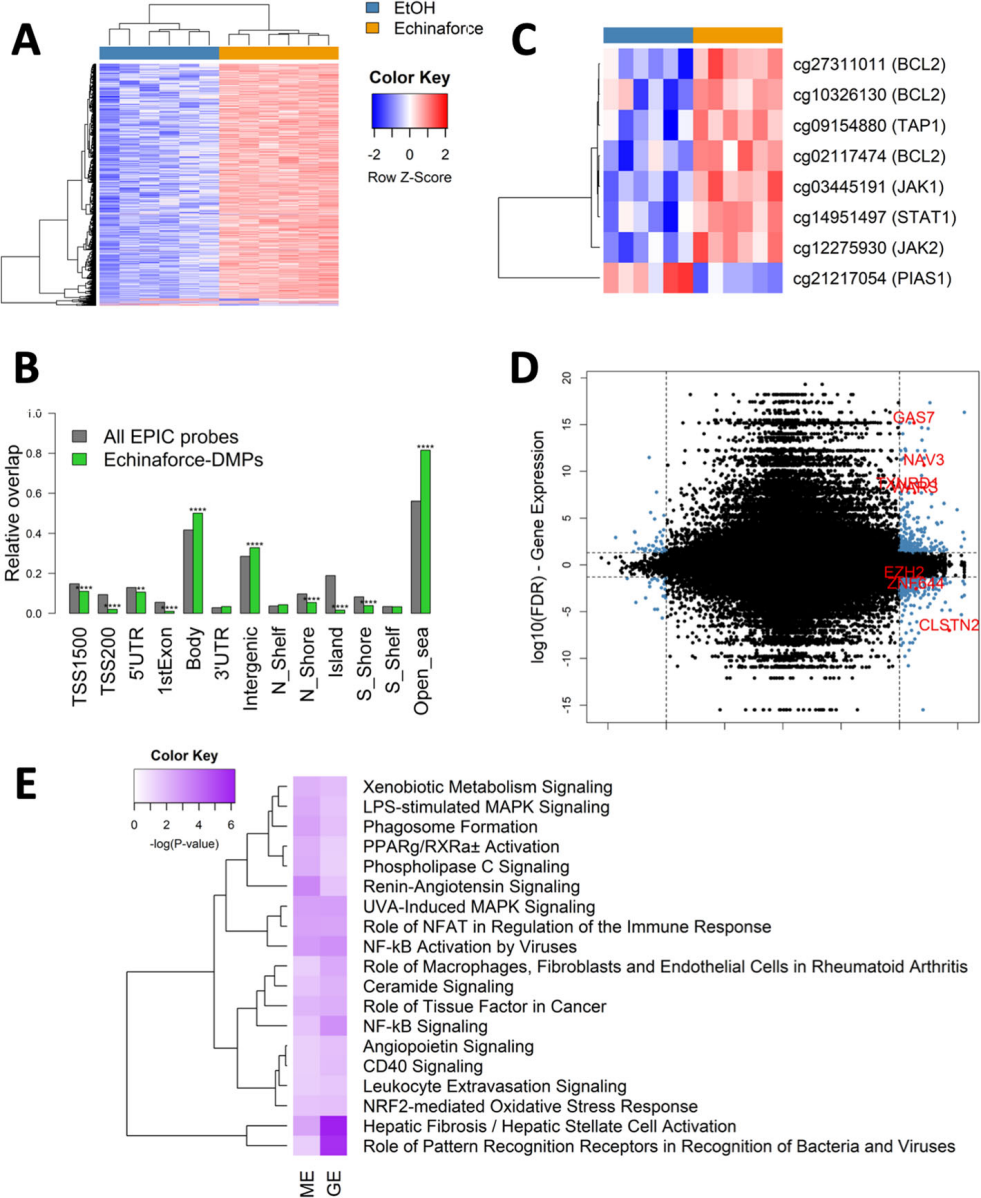
Echinaforce® treatment leads to global hypermethylation of CpG-poor gene bodies. **a** Heatmap showing the methylation values of differentially methylated probes upon treatment of THP1 cells for 48 h with Echinaforce® tincture (1%). Solvent (EtOH) controls are colored in blue and Echinaforce®-treated cells in orange. **b** Genomic enrichment of DMPs in different genomic regions. **c** CpG probes located in genes of the interferon signaling pathway which were differentially methylated (FDR < 0.1). * *P* ≤ 0.05, ** *P* ≤ 0.01, *** *P* ≤ 0.001, **** *P* ≤ 0.0001. **d** Starburst plot showing the genes both differentially expressed and differentially methylated. Each CpG-probe was mapped to its corresponding gene and the -log10(FDR) from the gene expression and DNA methylation analysis is displayed. The –log10(FDR) values of genes or CpG-probes with a negative LogFC or delta beta was multiplied by − 1 leading to positive values when logFC or delta beta was positive and negative values when logFC or delta beta was negative. CpG-probe – gene pairs which were differentially expressed (FDR < 0.05) and differentially methylated (FDR < 0.1) were colored in blue. The CpG-probe – gene pairs of which the absolute delta beta was higher than 0.05 and the absolute logFC higher than 0.4 were colored in red. **e** The IPA canonical pathways which were both significantly enriched in the gene expression and DNA methylation analysis

Epigenetics seems to be important in training immunity [60, 79] during monocyte differentiation and in the immunological memory of macrophages [80, 81]. Today, various bioactive phytochemicals have been identified which modulate inflammation through epigenetic reprogramming [82, 83]. Different phytochemicals and nutrients are known to change DNA methylation and histone modifications by directly influencing epigenetic enzymes or by interfering with the availability of the substrates/cofactors of these enzymes [84–86]. To assess whether the Echinaforce® induced changes in transcriptome profiles in THP1 cells are associated with DNA methylation changes, we measured complementary changes in DNA methylation profiles using the Illumina EPIC methylation array. Significant DNA methylation changes were observed following 48 h exposure to Echinaforce® (Fig. 6**a and Supplementary Table** **6**).

A total of 1875 CpG sites was found differentially methylated (FDR < 0.1) with a methylation difference of at least 5%. Typically, DNA methylation changes after short (24-72 h) exposure to phytochemicals and nutrients are much smaller than cancer associated DNA methylation changes in oncogenes or tumor suppressor genes which accumulate for many years in response to the microenvironment [48, 87, 88]. However, similar DMR effects sizes and cutoff (< 5%) were found to be biologically meaningful in various disease etiologies [42, 89, 90].

From the 1875 CpG sites identified, only 40 differentially methylated positions (DMPs) were hypomethylated whereas 1835 DMPs were hypermethylated. DMPs were mainly enriched in gene bodies, intergenic, and CpG-poor regions, while depleted in CpG islands, promoter, and enhancer regions (Fig. 6**b**). Only 1259 of the 1875 CpG-probes (67%) were located in a gene or 1500 bp upstream of a gene. Similarly, DNA methylation variation in the immune system was predominantly found at at CpG islands (CGI) within gene bodies, which have the properties of cell type-restricted promoters, but infrequently at annotated gene promoters or CGI flanking sequences (CGI “shores”) [91]. Subsequent IPA pathway enrichment analysis of the genes containing DMPs revealed inflammation or immunological diseases among others (**Supplementary Table** **6****).** Of particular interest, one of the top enriched pathways (‘Superpathway of Inositol Phosphate Compounds’) controls various epigenetic processes related to the interferon response [92–94].

Since both gene expression and kinase profiling both revealed the involvement of interferon signaling pathways, we also checked whether methylation of IFN pathway genes was affected by Echinaforce® treatment. Eight probes located in BCL2, JAK1, STAT1, PIAS1 and TAP1 did show an FDR < 0.1, with small methylation differences (between 1 and 3%) (Fig. 6**c)**. Whether these small methylation changes are sufficient to “train” the innate immune gene response needs further investigation [60, 61, 79].

Since most of the DMPs were located in intergenic regions and gene bodies, only a small subset of genes containing a DMP also resulted in a significant change in gene expression (Fig. 6**d**). Only seven genes were both differentially methylated and expressed, based on the significance criteria described above: i.e. Calsyntenin 2 (CLSTN2), Enhancer Of Zeste 2 Polycomb Repressive Complex 2 Subunit (EZH2), Growth arrest-specific protein (GAS)-7, neuron navigator (NAV)-3, Thioredoxin Reductase (TXNRD)-1, Tryptophanyl-tRNA synthetase (WARS) and Zinc Finger Transcription Factor (ZNF)-644. When using less stringent significance criteria, leaving out the effect size cutoff (logFC), 574 CpG site – gene pairs were found to be differentially expressed and methylated. Upon further comparing canonical pathways which are significantly enriched for both lists of differentially expressed genes and the list of differentially methylated genes, we identified 10 common biological processes (Fig. 6**e**). Remarkably, common pathways include NF-κB signaling (NF-κB activation by viruses, NF-κB signaling), MAPK signaling (LPS-stimulated MAPK signaling, UVA-induced MAPK signaling), and immune responses (i.e. Role of pattern recognition receptors in recognition of bacteria and viruses, Role of NFAT in regulation of the immune response, phagosome formation, CD40 signaling, leukocyte extravasation signaling).

### Echinaforce® treatment changes DNA repeat methylation and HERV transcription levels

**Fig. 7:**
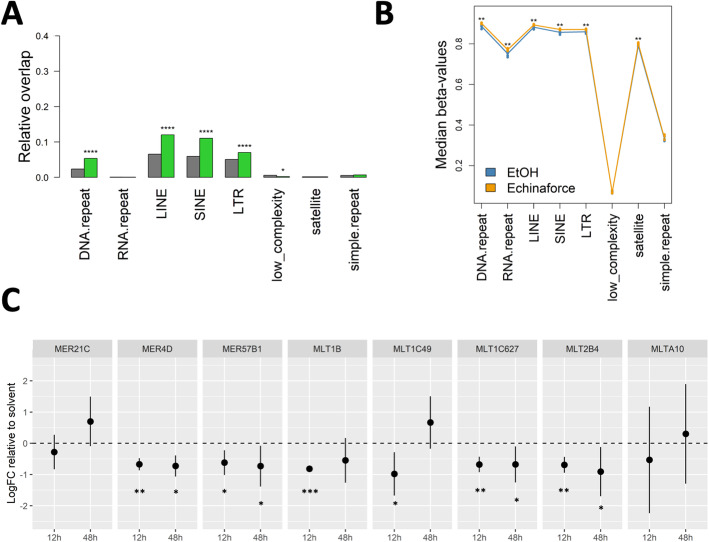
Echinaforce® treatment leads to global hypermethylation of intergenic repeat elements. **a** Genomic enrichment of DMPs in different repeat elements. **b** Global DNA methylation changes in different repeat elements. **c** HERV qPCR gene expression. THP1 cells were with Echinaforce® at 12 and 48 h (*n* = 3). Mean LogFC change relative to solvent control is represented together with 95% confidence interval. *: *P* ≤ 0.05, ** *P*: ≤ 0.01, *** *P*: ≤ 0.001 and **** *P*: ≤ 0.0001

DNA repeats and transposons require hypermethylation to maintain genomic instability and prevent transposition [95–99]. Interestingly, differentially methylated probes (DMPs) demonstrated a considerable enrichment in LINE, SINE and LTR transposon repeats, flanking endogenous retroviral sequences (HERVs) (Fig. 7**a-b**). This DMPs decreased transcription of MER4D, MER57B1, MLT1C627, MLT2B4 HERVs after 12 and 48 h Echinaforce® treatment, whereas MLT1B and MLT1C49 HERVs were only transiently repressed at 12 h (Fig. 7**c**). However, it remains unclear whether innate immune signaling (IFN response, chemotaxis, and immunometabolism) is driving HERV regulation or vice versa to mediate viral protection.

## Discussion

In this study, we applied for the first time a systems biology approach to characterize a possible mode of action of a standardized medicinal *Echinacea purpurea (*L.) Moench tincture Echinaforce®, which is widely used as a herbal remedy against respiratory tract infections. Microarray, QPCR, Western and multiplex immunoassays demonstrate that treatment of THP1 monocyte cells with Echinaforce® phytochemicals elicit time dependent gene expression changes in antiviral innate immunity signaling networks, involving tonic IFN (MX1, IFNβ, IFNγ, IFITM1, STAT1, STAT2) chemotaxis (IL8, CXCL10) and immunometabolic (ISG15, PKM2, SQSTM1) signaling pathways.

Most cells express a set of membrane and cytoplasmic receptors to detect viral RNA and DNA molecules: Pattern Recognition Receptors (PRRs). These receptors control innate immune signaling to activate the synthesis of interferons during a viral infection. In addition to pathogens, autophagy, metabolic and chemical stress, DNA damage, unfolded protein response, can also regulate innate immunity through cell-autonomous responses. Either IFN-inducible or constitutive, these processes aim to guarantee cell homeostasis or a biodefense mechanism against (non-self) hazardous molecules [100]. Of importance, these distinct constitutive cell-autonomous responses appear to be interconnected and can also be modulated by microbes, viruses and PRRs [101]. Our results suggest that Echinaforce® phytochemicals train innate immunity pathways via activation of interferon and chemokine gene expression. As such, secondary metabolite phytochemicals involved in plant immunity may prime evolutionary conserved innate immune responses across species [102–104].

For example, Echinaforce*®* treatment increases expression IFI27 and IFITM1, which both play critical roles in antiviral immunity and disease severity in respiratory disease [105–107]. Along the same line, transcriptional upregulation of the protein kinase receptor (PKR, EIF2AK2), a cytoplasmatic pattern-recognition receptor could be observed. PKR is known to transduce RNA helicase (MDA5) dependent virus signals for type I IFN induction [108]. Interferon regulatory factor 7 (IRF7) is another key protein found strongly upregulated. Transcription factors IRF7 together with IRF3 regulate expression of early type I IFN and other proteins involved in the innate antiviral immune response (*activation of IRF by cytosolic pattern recognition receptors*) [109] (Supplementary Tables 1, 2, 3, 4). Signal transduction via PKR occurs mainly via NFκB and MAPK pathways (*Role of PKR in Interferon induction and antiviral response*) [110]. Another important intracellular pattern-recognition receptor for viral RNA which was found to be upregulated by Echinaforce*®* was the RNA helicase MDA5 (IFIH1) [78]. Furthermore, upregulation of the NF-κB subunits RelB and NFKB2/p52 was observed, which can promote downstream production of innate immunity chemokines (*NF-κB activation by viruses, NF-κB signaling*) [111].

In line with our results showing activation of tonic IFN regulation of innate immunity gene responses, antiviral effects against influenza infection and activation of IFN pathways have also been demonstrated in vivo following Echinaforce® tincture treatment [10, 14, 20]. Along the same line, Echinaforce® treatment holds promise to reduce disease severity symptoms in SARS-CoV2 patients by strengthening impaired IFN specific innate immune signaling [64, 70, 71]. Our in vitro results are also in line with observations in human studies ex vivo*/*in vivo showing increased immunomodulating as well as chemotactic neutrophil effects following Echinaforce® treatment [10, 22, 23, 112]. For example, the antiviral ability of CXCL10 has been attributed to its chemoattractant effects which promote recruitment of natural killer cells [113–116] and neutrophils [113–116]. The latter illustrates that both neutrophils and inflammatory monocytes are intertwined in the immune system’s anti-viral response [113–116]. Similar results were previously obtained in murine dendritic cells, illustrating that Echinaforce® stimulates cell mobility and chemotaxis and alters expression of cell adhesion and motility genes [117]. Other studies showed that Echinaforce® may reverse the chemokine induction of virus-infected cells [11, 118–120]. Paradoxically, Echinaforce® may induce cytokine and chemokine expression in uninfected cells, but suppress their expression upon virus infection or LPS stimulation [29, 118–120]. Similarly, Echinaforce® increased the transcription of TNFα in human monocytes, but reduced the LPS-stimulated TNF-α protein production [19]. Although studies suggest that this stimulatory effect may be the result of bacterial-derived LPS and lipoproteins [26–29], our Echinaforce® tincture contains no polysaccharides, neither endotoxins. Altogether, the latter suggests that its immunomodulatory effects are due to the active compounds present in the formulation [12, 19]. Similar activation of IFN innate immunity and viral protection has been observed in presence of avocado and apple extract [121, 122]. Interestingly, in the latter case, effects were attributed to oligomeric proanthocyanidins and lost with their monomeric form [122].

With respect to immunometabolism, mitochondrial metabolism shows a remarkable sensitivity to chemokine and IFN signaling [123, 124]. For example, ISG15 is an interferon-stimulated, ubiquitin-like protein which regulates mitochondrial homeostasis and targets various proteins involved in catabolic autophagy metabolism in the mitochondria (mitophagy) during infection [125, 126]. Moreover, mitochondrial changes in immunometabolism (glycolysis, the tricarboxylic acid (TCA) cycle, the pentose phosphate pathway, fatty acid oxidation, fatty acid synthesis and amino acid metabolism) strongly contribute in (re) shaping immunity and production of neutrophil extracellular traps (NETs) [127–130].

Next, phosphopeptide based kinome activity analysis revealed Echinaforce® specific activation of innate immunity and IFN signaling via multiple kinases, including JAK1, TEC, p38 MAPK (MAPK11, − 12, − 13, and − 14), JNK (MAPK8, − 9 and − 10) and ERK1 kinases [131, 132]. JNK-STAT1 signaling induces various IFN responsive genes [133]. Moreover, JAK1 dependent regulation of downstream IFN and chemokine related gene expression after Echinaforce® treatment, could be reversed with the specific pharmacological JAK1 inhibitor filgotinib. TEC activation has important roles during innate immunity, i.e. IFN signaling via phosphorylation of JAK1 and JAK2 [131, 132], TLR signaling [134], assembly and activation of the caspase-8 inflammasome [135], macrophage survival [136], IL8 production [137], phagocytosis [138], and NFκB signaling [139]. p38 MAPK activation is involved in RIG-I dependent IFN signaling [140]. Various studies confirm involvement of these kinases in *Echinacea* biological action [19, 117, 141–144]. Alkylamides in the Echinaforce® tincture were found to be responsible for MAPK effects upon binding to CB2 receptors leading to increased cAMP, P38/MAPK and JNK signaling, NFκB and ATF-2/CREB-1 activation [19]. Similarly, lipophilic extracts of *Echinacea* promoted murine dendritic cell maturation and mobility via the modulation of JNK, P38 MAPK and NFκB pathways [117, 141, 142]. Another study demonstrated a JAK-STAT1 dependent antiviral response of *Tripterygium wilfordii* (Thunder of God Vine) via the quinone methide triterpene celastrol [145].

Finally, genomewide epigenetic analysis of DNA methylation changes following Echinaforce® treatment revealed almost 2000 DMP, enriched for immune disease and immunological pathways. Although the observed methylation changes are relatively small after 72 h treatment, cumulative effects can contribute in building an immune memory response by priming chromatin to mount faster and higher innate immune transcription upon re-stimulation of immune cells [103]. Besides the regulation of gene expression, DNA methylation is also involved in regulating alternative splicing, intron retention or promote cryptic transcription of non-annotated TSSs (TINATs) encoding immunogenic peptides which might prime an antiviral innate immune response [146–149]. As such, it appears that Echinaforce® treatment predominantly promotes epigenetic changes in innate immunity gene pathways and to a less extent of adaptive immunity responsive genes.

Besides, the higher global DNA hypermethylation observed after Echinaforce® treatment in LINE, SINE and LTR transposon repeats flanking endogenous retroviral sequences (HERVs), may be part evolutionary conserved (epi) genomic protective response against retrotransposition and viral infection [150, 151]. Similarly, IFN was shown to promote DNA methylation silencing of repeats and noncoding RNAs [39, 150, 152, 153]. Specific HERVs have been proposed to establish a protective effect against exogenous viral infections [154]. HERVs can act as IFN-inducible enhancers and have shaped the evolution of a transcriptional network underlying the IFN response [154–157]. Of particular interest, the MER41B family of ERV sequences contains a STAT1 binding site and regulates expression of IFN-γ–responsive genes, such as *absent in melanoma 2* (AIM2), and IFI6 [158, 159]. CRISPR-Cas9 deletion of a subset of these HERV elements in the human genome impaired expression of adjacent IFN-induced genes and revealed their involvement in the regulation of essential immune functions, including activation of the AIM2 inflammasome. Along the same line, DNA methylation inhibitors trigger an IFN response through viral mimicry via transcription of dsRNAs of repetitive elements from HERVs which can activate RIG-I and MDA5 PRRs [150, 151]. RNA transcripts of HERVs can be reverse transcribed to generate ssDNA or expressed to generate proteins with viral signatures, much like the pathogen-associated molecular patterns of exogenous viruses, which allows them to be detected by the innate immune system [160, 161]. In another example, silencing of the MLT1C49 HERV decreased expression of CXCL10 and CCL2 chemokines [162]. Finally, transcriptional changes of MLT1B and MER4D HERV transcription and innate immune signaling have also been described upon immunometabolic mitochondrial changes in protein kinase (PK)-M2 activity, which were counteracted by NFκB RelB [163]. From these examples, it appears that HERV regulatory sequences now constitute a dynamic reservoir of IFN-inducible enhancers fueling genetic innovation in mammalian immune defenses [158, 164, 165].

Previous studies showed that Echinaforce®, besides its immunomodulating activities is also very active as a virucidal agent against viruses with membranes, i.e. HSV-1, respiratory syncytial virus, all tested human and avian strains of influenza A virus, as well as influenza B virus [166]. Along the same line, *Echinacea* polyphenol quercetin was found to inhibit the entry of HIV-luc/SARS pseudotyped virus into Vero E6 cells [167]. Similar protective effects could recently also be observed in a reconstituted nasal epithelium cell culture system by exposing Echinaforce®-treated respiratory epithelium to droplets of HCoV-229E, SARS- or MERS-CoVs, imitating a natural infection [168]. In contrast Echinaforce® was found to be less effective against intracellular virus replication [168]. Consequently, virus already present within a cell could be refractory to the inhibitory effect of Echinaforce®, but virus particles shed into the extracellular fluids would be vulnerable. Therefore, the antiviral actions of the Echinaforce® may especially manifest during initial contact with the virus, i.e. at the inception of infection, and also during transmission of virus from infected cells.

## Conclusion

In conclusion, our systems biology approach revealed that Echinaforce® phytochemicals trigger multiple antiviral innate immunity pathways, involving tonic IFN signaling, activation of pattern recognition receptors, chemotaxis, immunometabolism and DNA hypermethylation of endogenous retroviral sequences. Further studies in preclinical respiratory infection models and double blind placebo-controlled intervention studies are needed to proof its prophylactic efficacy against common cold corona viruses (CoV), Severe Acute Respiratory Syndrome (SARS)-CoV, and new occurring strains such as SARS-CoV-2, with strongly impaired interferon (IFN) type I response and weak innate antiviral defense.

## Supplementary Information

**Additional file 1: Supplementary Table 1**: Differentially expressed probes (FDR < 0.05 and logFC > 0.4) after Echinaforce® tincture treatment.

**Additional file 2: Supplementary Table 2**: Enriched Ingenuity canonical pathways of differentially expressed genes after Echinaforce® tincture treatment.

**Additional file 3: Supplementary Table 3**: Ingenuity pathway enrichment analysis of diseases and biological functions of differentially expressed genes after Echinaforce® tincture treatment.

**Additional file 4: Supplementary Table 4:** STRING protein-protein-interaction plot and Metascape protein-protein-interaction MCODE network enrichment analysis of differentially expressed genes after Echinaforce® tincture treatment.

**Additional file 5: Supplementary Table 5**: PamGene upstream kinase analysis.

**Additional file 6: Supplementary Table 6**: Differentially methylated positions (FDR < 0.05 and |DeltaBetas| > 0.05).

**Additional file 7: Supplementary Table 7**: Enriched Ingenuity canonical pathways of differentially methylated genes after Echinaforce® tincture treatment.

## Data Availability

Data are available on request. Transcriptome and DNA methylation data were uploaded to the Gene Expression Omnibus (GEO) database and have accession number: GSE117904.

## Abbreviations

IFN: Interferon
ISG: Interferon stimulated genes
PTK: Protein tyrosine kinase
STK: Serine threonine kinase
JAK: Janus kinase
STAT: Signal transducer and activator of transcription factors
IRF: Interferon regulatory factor
CXCL: Chemokine (C-X-C motif) ligand (CXCL)
HERV: Human endogenous retroviruses
DEG: Differentially expressed genes
CCL: CC chemokine receptor ligand
IL: Interleukin
TF: Transcription factor
PPI: Protein-protein interactions
DMP: Differentially methylated position
DMR: Differentially methylated region
LINE: Long interspersed nuclear DNA repeat elements
SINE: Short interspersed nuclear DNA repeat elements
LTR: Long terminal DNA repeat
CGI: CpG island
